# Soluble plasma proteins ST2 and CD163 as early biomarkers of nephropathy in Swedish patients with diabetes, 15–34 years of age: a prospective cohort study

**DOI:** 10.1186/s13098-017-0240-2

**Published:** 2017-05-25

**Authors:** My Samuelsson, Jonatan Dereke, Maria K. Svensson, Mona Landin-Olsson, Magnus Hillman, on the behalf of the DISS Study group

**Affiliations:** 1 Department of Clinical Sciences, Diabetes Research Laboratory, Lund University, BMC, B11, 221 84 Lund, Sweden; 2 Department of Medical Sciences, Uppsala University, Uppsala, Sweden; 3 Department of Endocrinology, Skåne University Hospital Lund, Lund, Sweden

**Keywords:** Diabetes, Nephropathy, Biomarkers, Complications

## Abstract

**Background:**

The aim of this study was to investigate plasma levels of sST2 and sCD163 to determine whether they at an early stage could predict development of diabetic nephropathy and/or diabetic retinopathy in patients at clinical onset.

**Methods:**

Patients diagnosed with diabetes mellitus at age 15–34 years between 1987 and 1988 (n = 220) were included. Data such as BMI, smoking, HbA1c and islet cell antibodies were collected at time of diagnosis. Within the 10 year follow-up period, 112 patients (51%) developed following diabetes related complications; retinopathy (n = 91), nephropathy (n = 12) or both (n = 9). Plasma concentrations of sST2 and sCD163 were measured at time of diagnosis and levels compared between different complication groups.

**Results:**

Plasma levels of sST2 were significantly higher in patients who later developed nephropathy (n = 21; 1012 [773–1493] pg/ml) compared to those who did not (n = 199; 723 [449–1084] pg/ml; p = 0.006). A tendency for higher plasma levels of sCD163 was observed but not statistically significant (p = 0.058).

**Conclusions:**

sST2 and sCD163 show promise as potential biomarkers for the development of nephropathy already at clinical onset. sST2 and/or sCD163 could possibly be part of a biomarker panel aimed to find patients at high risk of developing nephropathy. Both markers need to be investigated in a larger prospective study.

## Background

Diabetes related long-term complications cause high economical and personal costs and impact quality of life. In many cases these long-term complications can be prevented or delayed if diagnosed and treated in a timely manner [1]. It is clear that in order to administer proper treatment early on, more effective predictive markers of long-term complications are needed. One complication of particular importance is diabetic nephropathy (DN), a severe microvascular disease, and one of the primary causes of morbidity and mortality amongst patients with diabetes mellitus [2]. Several factors related to DN development have been identified, such as hyperglycemia, hypertension, genetic predisposition and epigenetics, however a simple and effective predictive marker for DN is yet to be found [1, 3]. DN is currently treated with lifestyle interventions, intensive blood pressure- and glycemic control, and treatment of dyslipidemia. Despite available therapies there is a progression of the disease [4, 5]. In addition, some patients develop DN rapidly even with modest hyperglycemia while others never develop DN, in spite of poor glycemic control. This suggests genetic traits and molecular mechanisms of the disease that are not yet fully understood.

Diabetic nephropathy can be classified after level of severity; incipient nephropathy (microalbuminuria) and overt nephropathy (macroalbuminuria), the latter being more severe and strongly related to development of renal impairment and end-stage renal disease (ESRD) [6]. Increased urinary excretion of albuminuria is an early clinical manifestation of renal damage or dysfunction in diabetes and is also an important risk factor for progression of renal impairment and development of ESRD [7] as well as an established risk factor for cardiovascular disease (CVD) and all-cause mortality [8]. Microalbuminuria is defined as a urine albumin excretion rate (UAER) of 20–200 µg/min and macroalbuminuria as an UAER of >200 µg/min. Microalbuminuria is generally a sign of glomerular damage and macroalbuminuria is usually accompanied by clinical manifestations of renal impairment such as reduced glomerular filtration rate (GFR) [9] as well as more prominent structural histological lesions in the kidney [10].

The most commonly used biomarkers for identifying renal disease progression today are serum creatinine and estimated GFR (eGFR). The precision is however low and there is need for more sensitive markers for early prediction of nephropathy. Several novel tubular and inflammatory biomarkers have been evaluated as potential predictors for nephropathy, most of which have been investigated in early kidney damage [11]. A couple of urinary tubular biomarkers has been reported to be increased in patients with type 1 diabetes several years before development of DN. Further studies in both urine and blood are required to confirm these findings and to study if the results persist in patients with type 2 diabetes [12, 13].

Diabetic retinopathy (DR) is another diabetes related long-term microvascular complication. The retinal vasculature is sensitive to hyperglycemia which induces oxidative stress, an increase in advanced glycation end products (AGEs) and activation of protein kinase C (PKC) amongst other destructive processes [14]. These underlying changes cause microaneurysms, macular edema and neovascularization impairing vision [15].

Due to the complexity of complication development it is probable that a future biomarker panel consisting of several different proteins and miRNAs could yield better precision than the use of single biomarkers. Thus there is a need to identify potential candidates.

Soluble ST2 (sST2) is a free form of the membrane bound ST2, found on the surface of Th2-cells, mast cells and basophil granulocytes. ST2 is an interleukin-33 (IL-33) receptor which upon binding induces Th2 activity and increases Th2 inflammatory responses by inducing chemotaxis in Th2-cells as well as the release of Th2-associated cytokines [16]. Presumably sST2 acts as a decoy receptor for IL-33, preventing it from binding to membrane bound ST2 and thereby reducing the biological effect of IL-33 [17]. Plasma levels of sST2 reportedly correlate with diabetic markers such as triglycerides, hepatic function and plasma glucose as well as incidence of diabetes mellitus [18, 19]. Thus, sST2 may play part in the inflammatory process of diabetes mellitus, although the mechanisms behind this are not yet fully understood. Levels of sST2 have been associated with cardiovascular complications and mortality [20, 21] and suggested to have a prognostic value as risk predictor in patients with ambulatory chronic heart failure [22].

Soluble CD163 (sCD163) is the shedded ectodomain of membrane bound CD163, an endocytic receptor found on some monocyte and macrophage populations [23]. The biological function of sCD163 is not entirely understood, although levels of sCD163 rise markedly during inflammation and insulin resistance [24] making it a promising biomarker for macrophage involvement in prediabetes [25]. Furthermore, sCD163 interacts with a ligand known as Tumor Necrosis Factor Weak Inducer of Apoptosis (TWEAK) which have been associated to obesity and type 2 diabetes [26], but also to tissue regeneration after ischemic injury [27]. Increased expression of TWEAK in vitreous samples have been reported in patients with DR [28], however the possible interaction with sCD163 was not investigated. High levels of sCD163 results in an increased risk of type 2 diabetes development [29], maybe as a result of macrophage infiltration to adipose tissue and liver.

The aim of this study was to assess plasma levels of sST2 and sCD163 to determine whether they are associated with development of diabetes related long-term complications (DN and DR) in patients with onset of diabetes as young adults. If so, sST2 and/or sCD163 might serve as early biomarkers or to be included in a panel of biomarkers identifying patients at high risk for development of diabetes related complications and enable a closer monitoring of high risk patients as well as facilitate development of better treatment options to delay or prevent onset of complications.

## Methods

### Study population

Subjects included in the study were diagnosed with type 1 (n = 131) or type 2 diabetes (n = 89) mellitus by their reporting physician according to World Health Organization criteria [30]. All subjects were part of the Diabetes Incidence Study in Sweden (DISS) [31]). A total of 220 participants were included, all diagnosed with diabetes mellitus in 1987–88 at an age of 15–34 years. Out of these patients, 112 (51%) developed the following diabetes related complications within the 8 year follow up period; DR (n = 91), DN (n = 12) or concomitant DR and DN (n = 9). The remaining 108 developed neither of the complications and were used as a control group. In this study, the term DN refers to persistent micro- or macroalbuminuria, thus incipient or manifest DN. Persistent microalbuminuria (incipient nephropathy) and macroalbuminuria (overt nephropathy) are both referred to as DN [32]. Identification of patients with DN was performed in two steps as previously reported and patients were not categorized as having DN if other causes for albuminuria were found. The selection and classification of the study population has been described in greater detail elsewhere [31].

### Sample handling

Blood samples were collected in tubes containing EDTA at time of clinical diagnosis of diabetes mellitus. Samples were sent to the laboratory from health care centers and hospitals in Sweden by ordinary mail. Plasma was separated by centrifugation at 2000×*g* and stored in −20 °C until use. HbA1c levels were measured at local hospitals by ion chromatography and presented in NGSP % followed by International Federation of Clinical Chemistry (IFCC) units (mmol/mol). Both sCD163 and sST2s are verified as stable in frozen plasma samples and reportedly robust to thawing [29, 33].

### Laboratory analyses

Plasma concentrations of sST2 and sCD163 respectively were measured in duplicates by the use of sandwich enzyme-linked immunosorbent assay (ELISA) (R&D Systems, Minneapolis, Minnesota, USA) DuoSet kit and supplemental DuoSet Ancillary Reagent Kit 2 (R&D Systems, Minneapolis, Minnesota, USA) according to manufactures instructions. Two in-house plasma controls were added to each run. The sST2 kit used has a reported lower detection limit of 32 pg/ml [33]. The inter assay variability was calculated to 11.6% for sST2 and 9.7% for sCD163. Samples were diluted, sST2 1:3 and sCD163 1:200 respectively. All samples were added in duplicates, subjects and controls alternated on each plate to reduce inter-assay variability. Optical density (OD) was measured at 450 and 580 nm with a FLUOstar OPTIMA microplate reader (BMG LABTECH, Ortenberg, Germany). A four-parametric logistic regression standard curve was used to read the concentrations.

### Statistical analyses

D’Agostino-Pearson test was used to test for normal distribution of data. Results are presented as mean ± SD when normality was accepted and median followed by interquartile range (Q1–Q3) when normality was rejected. Differences between groups were tested with Student’s *t* test for normally distributed data otherwise Mann–Whitney U-test or Kruskal–Wallis H-test were used. Differences in observed and expected frequencies between groups were calculated using χ^2^-test. Correlations between variables were determined using Spearman’s rank correlation test. p values below 0.05 were considered as statistically significant. All statistical analyses were performed using MedCalc^®^ version 14.12.0 for Windows (MedCalc Software, Ostend, Belgium).

## Results

Clinical and laboratory data in study participants are summarized in Table 1.

**Table 1:** Biochemical and clinical data for study participants, aged 15–34 at onset of diabetes, with or without development of diabetic nephropathy (DN) within 10 years after onset of diabetes

	No nephropathy (n = 199)	Nephropathy (n = 21)	p value
sST2 (pg/ml)	723 [449–1084]	1012 [777–1493]	0.006
sCD163 (ng/ml)	287 [221–386]	346 [299–450]	0.058
BMI (kg/m^2^)	25.1 ± 3.5	25.1 ± 4.1	0.911
Age (years)	25.0 ± 5.5	26.7 ± 6.1	0.181
HbA1c (mmol/mol (%))	66.1 ± 15.6 (8.2 ± 1.4)	74.0 ± 17.2 (8.9 ± 1.6)	0.031
Sex (men/women)	118/81	15/6	0.397
Type 1/type 2 diabetes	122/77	9/12	0.160

Patients who developed DN within the first 10 years after onset of diabetes mellitus had significantly higher plasma level of sST2 (n = 21; 1012 [773–1493] pg/ml) at time of onset of diabetes mellitus when compared to patients that did not develop DN (n = 199; 723 [449–1084] pg/ml; p = 0.006). This increase in sST2 levels was even more prominent when taking the severity of DN into account since patients that developed macroalbuminuria had the highest plasma levels (n = 5; 1629 [1416–1849] pg/ml) compared to patients that developed microalbuminuria (n = 16; 917 [704–1108] pg/ml; p = 0.002). No differences in sST2 levels at onset of diabetes were found between participants who later developed DR and those who did not (p = 0.54). Plasma levels of sST2 were significantly higher in men (900 [597–1216] pg/ml) than in women (596 [342–777] pg/ml; p < 0.0001). No differences in sST2 levels were found between smokers and non-smokers (p = 0.65) or between patients with type 1 diabetes and type 2 diabetes (p = 0.98). No statistically significant correlation was found between sST2 and BMI although a tendency was observed (rs = 0.13; p = 0.06). No correlation between plasma levels of sST2 and age (rs = −0.08; p = 0.22) or HbA1c (rs = −0.0006; p = 0.99) was found.

Plasma levels of sST2 and sCD163 levels were strongly correlated (rs = 0.23; p = 0.002). There was also a tendency of higher sCD163 levels in patients that later developed DN when compared to those who did not (p = 0.058). Levels of sCD163 did not correlate significantly with age (rs = −0.05; p = 0.51), BMI (rs = 0.08; p = 0.23) or HbA1c levels (rs = −0.05; p = 0.51), nor did it differ between smokers and non-smokers (p = 0.74), men vs. women (p = 0.45) or between patients with type 1 and type diabetes (p = 0.27).

Levels of HbA1c differed significantly (p < 0.0001) between controls [60 mmol/mol (7.6%)], and patients with DR only [72.5 mmol/mol (8.8%)], and concomitant DR and DN [81 mmol/mol (9.6%)], but not DN only [61.5 mmol/mol (7.8%)] (p = 0.49). No statistically significant differences between the four complication groups regarding BMI (p = 0.84), sex (p = 0.44), age (p = 0.11), smoking (p = 0.95) or diabetes classification (p = 0.28) were observed (Table 1), thereby reducing their possible confounding effects.

Patients diagnosed with type 1 diabetes had lower BMI (24.5 ± 3.1) compared to patients diagnosed with type 2 diabetes (25.9 ± 3.9) (p = 0.005) as expected. Patients diagnosed with type 1 diabetes were also slightly younger (24.3 ± 5.4 years) compared to type 2 diabetes (26.4 ± 5.8 years; p = 0.008). Plasma levels of sST2 and sCD163 did not differ between patients diagnosed with type 1 and type 2 diabetes. No difference in complication development was found between patients with type 1 and type 2 diabetes (p = 0.86). A frequency of 6.9% of patients with type diabetes and 13.5% of patients with type 2 diabetes developed DN (p = 0.11).

## Discussion

The main and novel finding in this study is that plasma sST2 levels were significantly higher and at the time of onset of diabetes in subjects who early on, within 10 years, developed nephropathy. Levels of sCD163 were also higher in the same group although did not reach statistical significance. Furthermore, levels of sST2 increased significantly with degree of albuminuria and thus the severity of nephropathy. This significant difference in plasma sST2 levels remained after adjusting for sex, age and BMI. However, levels of sST2 did not differ between patients developing DR or complications in general when compared to controls. Thus, sST2 could be a promising candidate in a future panel identifying patients at high risk of developing particularly renal involvement and diabetes nephropathy.

A strength with the study is that we have a well-defined cohort with young adult subjects and a long follow up time of 10 years making it possible to longitudinally identify complication development. Another strength is that we used commercially available assays with high sensitivity and low variation. A limitation of the study is that relatively few patients developed nephropathy within the follow up period which impacts the statistical power. Most diabetes related complications, DN in particular, occur 15–25 years after onset of diabetes [34]. It is thus possible that the patients in this study may be a subgroup of patients with early onset of nephropathy.

Plasma sST2 levels were not significantly different between men and women, type 1 and type 2 diabetes or smokers and non-smokers. The fact that sST2 levels were not associated with HbA1c levels, in combination with the high stability of sST2 in plasma and its general robustness makes sST2 a promising predictive biomarker for the development of DN. This is highly interesting since it raises the question of how the pathogenesis of DN and DR may differ from each other, as well as the potential role of sST2 in DN pathogenesis. This is also supported by the fact that not all patients with DN have concomitant retinopathy. This might seem like an unexpected finding, but it is well in line with previous studies [35, 36].

Levels of HbA1c were found to be significantly higher in patients who within 10 years developed diabetes related complications, DN and DR taken together. This is expected since HbA1c is a well-known predictive marker for development diabetes-related complication [34, 37]. Interestingly, subjects developing DN without concomitant DR did not have higher HbA1c levels compared to the control group. Patients with DR had significantly higher HbA1c levels when compared to the control group and DN only. Previous studies have found significantly higher HbA1c levels in patients developing long-term complications such as DN when measuring long term mean HbA1c from onset of diabetes, or at the time of onset of DN [34, 37, 38]. This suggests that sST2 and HbA1c might complement each other as predictive markers. Analyzing both markers simultaneously may enable a higher precision [39].

This study found a tendency of difference in sCD163 plasma levels between patients that did and did not develop nephropathy during the follow up period. Determining the relevance of this tendency would require further studies in larger populations. Previous studies have found associations between sCD163 levels and onset of type 2 diabetes [29] as well as its ligand sTWEAK in type 1 diabetes and complications [40]. To our knowledge associations between sCD163 and DN/DR have not previously been investigated or described.

We found no difference in plasma levels of sCD163 between men and women, type 1 and type 2 diabetes or smokers and non-smokers. Neither was any correlation between sCD163 levels and age or HbA1c found. There was no statistically significant correlation between sCD163 and BMI. This may at first glance seem surprising since high BMI often correlate with higher percentage of body fat, which in turn is associated with higher macrophage infiltration to fatty tissues as well as inflammation and macrophage activation [25, 41]. However, since our study population constitutes of younger individuals with type 1 diabetes being more common than type 2, the inflammatory mechanism behind the release of sCD163 might be different and the association with BMI of less significance.

Additional larger and prospective longitudinal studies on sST2 and sCD163 levels and development of nephropathy are needed, in both blood and urine. Preferably over a longer follow up period are needed to prove the correlation. We observed that 6.9% of the patients with type 1 diabetes and 13.5% of patients with type 2 diabetes developed DN. Although not reaching statistical significance this difference should be investigated in a larger cohort of patients at different ages. Additionally, there is room for further explorations regarding sST2’s possible role in the pathogenesis of DN as well as a possible target for potential therapies.

## Conclusions

In conclusion, there was a strong association between higher plasma levels of sST2 at clinical onset and development of DN within 10 years. With sST2 being highly stable and easily quantified it holds promise to be included in a biomarker panel for later DN development but have far too limited sensitivity and specificity to be used by its own. It is probable that more subjects would develop DN within a longer follow up period which might increase the sensitivity and specificity of the marker. It would therefore be of interest to study the predictive properties of sST2 on a later onset of complication development. To our knowledge, this is the first study in which any association between levels of sST2 or sCD163 with later development of DN has been assessed.
